# An Evolving Challenge: Fungal Infections in the Contemporary ICU

**DOI:** 10.3390/jof12030197

**Published:** 2026-03-09

**Authors:** Ayman O. Soubani

**Affiliations:** Division of Pulmonary, Critical Care and Sleep Medicine, Wayne State University School of Medicine, Detroit, MI 48201, USA; asoubani@med.wayne.edu

Fungal infections have become an integral and increasingly complex component of modern intensive care medicine. Improvements in critical care survival have expanded the population of patients exposed to prolonged mechanical ventilation, broad-spectrum antimicrobial therapy, central venous access, extracorporeal organ support, and immunomodulatory agents, all of which contribute to profound alterations in host immunity. Fungal infections in critically ill patients are common and are no longer limited to those who are immunocompromised. They are also associated with excess mortality, prolonged length of stay, and substantial healthcare utilization, particularly when diagnosis and initiation of appropriate antifungal therapy are delayed.

Invasive candidiasis remains the most frequently encountered fungal infection in critically ill patients and constitutes the majority of fungal bloodstream infections in the ICU. While *Candida albicans* historically predominated, there has been a sustained global shift toward non-albicans species, including *Candida glabrata*, *Candida parapsilosis*, *Candida tropicalis*, and, more recently, the multidrug-resistant *Candida auris*. This epidemiologic evolution has significant therapeutic and stewardship implications, given species-specific differences in virulence, antifungal susceptibility, and propensity for nosocomial transmission. It is currently recommended to use an echinocandin as first-line empiric therapy for most ICU patients with suspected invasive candidiasis, while highlighting the importance of early de-escalation based on species identification and susceptibility testing [1].

In parallel, invasive aspergillosis has emerged as a major cause of morbidity and mortality in the ICU beyond its traditional association with neutropenia and hematologic malignancy. Critically ill patients with acute respiratory distress syndrome (ARDS), chronic obstructive pulmonary disease, liver failure, or prolonged corticosteroid exposure are now recognized as being at substantial risk [2]. Influenza-associated pulmonary aspergillosis (IAPA) and COVID-19-associated pulmonary aspergillosis (CAPA) have been highlighted by recent respiratory viral pandemics and reflect the role of severe respiratory viral infections and critical illness in predisposing to invasive aspergillosis [3]. Mortality in these populations remains high and is compounded by diagnostic uncertainty and delayed initiation of mold-active antifungal therapy.

Other rare fungal infections that may be associated with critical illness and require ICU care include mucomycosis in severely immunocompromised patients and those with diabetic ketoacidosis. Pneumocystis jirovecii in patients with HIV and other immunosuppressive conditions is another fungal infection that may be associated with acute respiratory failure and the need for invasive mechanical ventilation. Endemic fungi such as histoplasma and blasomyces are rare but may also be encountered.

Reported incidence rates of invasive fungal infections in the ICU vary widely according to case mix, surveillance intensity, and geographic region, but contemporary studies estimate that approximately 3–10% of critically ill patients develop these types of serious infections, with higher rates in selected populations, such as those with severe viral pneumonia or complicated intra-abdominal infections [4]. Across etiologies, mortality remains unacceptably high and is consistently linked to delays in appropriate antifungal treatment, reinforcing guideline recommendations for early therapy in high-risk patients while avoiding indiscriminate antifungal use [1,5].

The timely diagnosis of fungal infections in critically ill patients remains a central challenge. Clinical features are nonspecific and frequently indistinguishable from bacterial sepsis or sterile inflammatory syndromes. Conventional culture-based diagnostics lack sensitivity and are often delayed, while differentiation between colonization and invasive disease—particularly for *Candida* in respiratory or urinary specimens and *Aspergillus* in airway samples—remains problematic. Multiple diagnostic criteria have been suggested for the diagnosis of IFI in critically ill patients that endorse the use of non-culture diagnostics, including β-D-glucan, galactomannan, and molecular assays, as adjunctive tools, but acknowledge important limitations in ICU populations, including false positivity related to renal replacement therapy, antibiotic exposure, mucosal injury, and critical illness itself [6].

Therapeutic management is further complicated by altered pharmacokinetics in critical illness, organ dysfunction, extracorporeal support modalities, and clinically significant drug–drug interactions. Triazole antifungals require careful attention to absorption, metabolism, and therapeutic drug monitoring, while emerging resistance among *Candida* spp. and azole-resistant *Aspergillus* increasingly constrain treatment options. These challenges underscore the importance of ICU-specific antifungal stewardship strategies that integrate local epidemiology, rapid diagnostics, and individualized pharmacologic optimization.

Against this backdrop, future advances in the management of ICU-associated fungal infections will depend on improved early diagnostic strategies, refined risk stratification, and the development of novel antifungal agents with activity against resistant pathogens. The integration of predictive models, host immune biomarkers, and real-time surveillance data may allow for a transition from empiric to precision-guided antifungal therapy.

This Special Issue of the *Journal of Fungi* presents a synopsis of fungal infections in intensive care medicine, with original research and review articles that primarily focus on the two main severe fungal infections: candida and Aspergillus. Research papers on candida discuss resistance patterns, infection in cardiothoracic ICU, and the association with severe COVID infection. Another paper provides insight into the epidemiology and outcomes of invasive aspergillosis in patients with liver failure. The review papers discuss the state of the art in terms of the risk factors, diagnosis and management of these serious infections in critically ill patients.
